# The Modulated Role of *Toxoplasma gondii* on Eosinophils in Psychiatric Disorders after Cannabis Cessation

**DOI:** 10.3390/pathogens12111333

**Published:** 2023-11-09

**Authors:** Bruno Romeo, Valentine Lestra, Catherine Martelli, Ammar Amirouche, Amine Benyamina, Nora Hamdani

**Affiliations:** 1 Department of Psychiatry and Addictology, Paul Brousse Hospital, APHP, 94800 Villejuif, France; valentine.lestra@aphp.fr (V.L.); catherine.martelli@aphp.fr (C.M.); ammar.amirouche@aphp.fr (A.A.); amine.benyamina@aphp.fr (A.B.); 2 Unité de Recherche UR, Psychiatrie-Comorbidités-Addictions (PSYCOMadd), Paris Saclay University, 94800 Villejuif, France; nora_psy@yahoo.fr; 3 Institut National de la Santé et de la Recherche Médicale U1299, Research unit, NeuroImaging and Psychiatry, Paris Sud University-Paris Saclay University, Paris Descartes University, Digiteo Labs, 91190 Gif-sur-Yvette, France; 4 Cédiapsy, 75006 Paris, France

**Keywords:** inflammation, *T. gondii*, cannabis use, eosinophils counts

## Abstract

The aim of our study was to evaluate the impact of *T. gondii* status on eosinophils count (EOS), the eosinophil-to-lymphocyte ratio (ELR), and the eosinophil-to-neutrophil-to-lymphocytes ratio (ENLR) before and after cannabis cessation in patients with psychiatric disorders. One hundred and eighty-eight patients were included in the study. *T. gondii*, EOS, ELR, ENLR, and urinary cannabis were measured at baseline and after 4 weeks of cannabis cessation. Highest levels and increase of PNE (*p* = 0.02), ENLR levels (*p* = 0.031) and highest level of ELR (*p* = 0.03) were found in patients after cannabis cessation only in patients positive for *T. gondii* serology (Toxo+ group). At four weeks, significant interactions between cannabis and *T. gondii* status for EOS (*p* = 0.038), and for ENLR (*p* = 0.043) levels were found, as well as for the evolution between baseline and 4 weeks for ENLR level (*p* = 0.049). After cannabis cessation, we found a positive correlation between negative symptoms and EOS levels at 4 weeks in the Toxo+ group. This study shows that the increase of inflammation after cannabis cessation might be modulated by *T. gondii* seropositivity status in patients after cannabis cessation.

## 1. Introduction

*Toxoplasma gondii* is an intracellular parasite which affects approximately 30% of humans worldwide, with a higher heterogeneity depending on geographic location [1,2]. Indeed, the seroprevalence in the USA or the UK was estimated between 8 and 22% but in central and south America and Europe, the estimates range from 30 to 90% [3]. There is a higher prevalence of toxoplasma among psychiatric populations. A recent meta-analysis found a mean seroprevalence of 37% and 28%, respectively, in patients with bipolar disorder and in healthy controls [4] However, considering single studies, the seroprevalence can reach 76.9% in France [5]. Interestingly, the geographic variations of the seroprevalence was more important in healthy controls than in patients with psychiatric disorders [4]. Unfortunately, few studies have investigated the highest seroprevalence of *T. gondii* in addictive disorders, except a meta-analysis [6].

The hypothesis of immuno-inflammation induced by *T. gondii* could explain the particular link with psychiatric disorders and addictions [7,8]. The secretion of pro-inflammatory cytokines following *T. gondii* infection (such as IL-6, IL-1B and TNF-a) could affect the microglie and thereby affect the neurotransmitter secretion of serotonin and dopamine [9]. A link with cognitive deterioration has therefore been raised, as cognitive impairment (processing speed, working memory, executive functions) was found among healthy and psychiatric populations [1,10] which can be correlated to the degree of inflammation among patients [10,11].

The vulnerability-stress-inflammation model in psychiatric disorders has been largely described [12,13,14]. Notably, an activation of the innate system illustrated by an increase level of cytokine levels, such as Il-6, TNF-a, has been found among patients with schizophrenia, bipolar disorder or major depressive episode [12,13]. Further, the highest levels of leukocytes, monocytes and neutrophils, but also neutrophils-to-lymphocytes ratios (NLR), have been found for schizophrenia or first-episode psychosis [15] and bipolar disorder (mania/euthymia) [16], compared to controls [17] with inconsistent data regarding monocytes-to-lymphocytes ratios (MLR) [15]. In contrast to the innate system, the adaptative immunity in schizophrenia seem to be less involved compared to major depressive episodes or bipolar disorder [18,19,20,21,22].

Substance use disorders have an impact on inflammatory markers [23]. Some studies suggested that cannabis use was associated with lower inflammation in patients with psychiatric disorders (IL-6, IFN g and CRP) [24,25,26] However, these suggestions were made with inconsistant data [27,28]. One study found that cannabis use was not associated with specific inflammatory profiles, but a composite score representing the systemic inflammation state might moderate the cannabis–psychosis association in the first-episode of psychosis patients [29]. Finally, an increase of leucocytes, lymphocytes and monocytes was found in patients with psychosis [28].

Eosinophils may act as a key player of the innate and adaptive immune response [30]. Eosinophils play a pro-inflammatory and destructive role regarding the Th2 immune response triggered by allergic or parasitic infection. In addition, eosinophils also play a role in the adaptive system as non-professional, antigen-presenting cells in response to allergens or parasites, or by coordinating and regulating T lymphocytes and dendritic cell action [29].

Moreover, the eosinophil/lymphocyte ratio and eosinophil/neutrophil/lymphocytes ratio have been proposed as indicators of systemic inflammation in patients with cancer or with autoimmune rheumatic diseases [31]. They found that the highest ratios are associated with poorer prognosis [32]. These associations have also been found among smokers in psychiatric populations, as compared to non-smokers [31] and alcohol use disorder patients with comorbid bipolar disorder [33].

Eosinophils are thought to be effector cells in the body’s defense against parasitic infections, and the mechanism of action may differ depending on the parasite [34], but to our knowledge, no study has investigated the link between eosinophils, *T. gondii* serological status and cannabis use and cessation.

The aim of our study is to evaluate the impact of *T. gondii* serological status on eosinophils count (EOS), the eosinophil/lymphocyte ratio (ELR) and the eosinophil/neutrophil/lymphocyte ratio (ENLR) before and after cannabis cessation in patients with psychiatric disorders. The second objective of our work is to study the associations between these ratios, *T. gondii* status and clinical symptoms.

## 2. Materials and Methods

### 2.1. Participants

This retrospective study included one hundred and eighty-eight inpatients in the psychiatry and addictology department at Paul Brousse Hospital (Paris) between July 2019 and November 2022. The inclusion criteria were (i) inpatients during an acute phase of a psychiatric disorder, as defined by the international classification of diseases (ICD) 10 classification (F10-99), in which the diagnosis was performed by the psychiatrists in charge of the patients and was extracted from hospitalization records; and (ii) being over 18 years old, in which xclusion criteria included neurological disorders (F00 to F10 ICD 10 diagnosis) and use of anti-inflammatory treatments. In this non-interventional retrospective cohort study, only existing data from routine care were added and utilized. According to the Jarde law (2012), which modifies the public health law (2004), complete information was given to all patients on the possible retrospective use of their routine care data for research purposes. All patients were informed that they could refuse the study without consequences on the care provided. However, none of the included patients refused. This MR-004 non-interventional study was conducted in accordance with the modified 1978 data-processing and freedom law, the declaration of Helsinki, and was approved by the French Data Protection Authority (N°1980120), which is a French institution devoted to the protection of participants.

### 2.2. Data Collected

Socio-demographic data were collected, which included comorbidities, any undergoing treatments, smoking status, and body mass index (BMI). The Positive and Negative Syndrome Scale (PANSS) scale [35] was assessed by trained psychiatrists. These PANSS scores were only extracted for patients with psychosis (i.e., diagnosis of schizophrenia, schizoaffective disorder, other psychotic disorder, bipolar disorder or major depressive disorder with psychotic symptoms). At baseline, *T. gondii* serology was assessed by a chemiluminescence enzyme immunoassay (Liaison ^®^ XL DiaSorin, Vercelli, Italy), which has a sensitivity at 99.3% and a specificity at 96.8% [36]. White blood cell (WBC) results, assessed by flow cytometry (Symex XE-5000, Kobe, Japan/Advia 2120i^®^, Eschborn, Germany), were collected at baseline and 4 weeks later. Urinary cannabis was also collected upon admission and 4 weeks later using an immuno-enzymatic method (C800 Abbott ^®^, Abbott Park, IL, USA). All biological samples were analyzed in the laboratory of Paul Brousse Hospital. All data were extracted from the computerized medical records and stored in an anonymized file in accordance with the French Data Protection Authority declaration.

### 2.3. Data Analyses

Analyses were performed with Jamovi (Version 2.2), a graphic user to the R statistical analysis software for scientific medical publications (Available on: https://www.jamovi.org). To compare EOS, ELR, and ENLR levels between cannabis user (THC+) and non-users (THC-) among positive patients for *T. gondii* serology (Toxo+) vs. negative patients (Toxo−), a Shapiro–Wilk test was performed to confirm the normal distribution of samples. For samples including more than 30 patients with a normal distribution of parameters, a t-test was used to compare THC+ versus THC- in patients Toxo+ and in patients Toxo−, and a paired sample t-test was used to compare the intra-group changes. We used a Mann–Whitney non-parametric test and a Wilcoxon test to compare the intra-changes in small samples (<30 patients), and/or if the data distribution was not normal. For categorical variables, a Chi-square test was used. To assess the association between EOS, ELR, ENLR and sociodemographic or PANSS score, Spearman or Pearson correlations were computed.

Two multivariate analyses were performed using linear regression. At baseline and at 4 weeks, we included age, gender, use of antipsychotics (Yes/No), use of mood stabilizers (Yes/No), use of antidepressants (Yes/No), use of anxiolytic (Yes/No), BMI, smoking status (Yes/No), cannabis status (Yes/No), presence of illness that may impact inflammation (Yes/No), and diagnosis of psychosis (Yes/No).

To evaluate the interaction between *Toxoplasma gondii* and cannabis status on EOS, ELR and ENLR, a general linear model was performed at baseline and 4 weeks using the same confounding factors used in the linear regression.

## 3. Results

### 3.1. Description of the Population

One hundred and eighty-eight inpatients were included in this study (106 Toxo+, 82 Toxo−) at baseline, and one hundred and nineteen attended the follow-up at 4 weeks (58 Toxo+ vs. 61 Toxo−). Table 1 describes the main characteristics of the sample. Toxo+ patients were older than Toxo− patients (*p* < 0.01). There were also more smokers in the Toxo+ group (*p* < 0.01). No difference was found for BMI, gender, cannabis status, diagnosis and PANSS score at baseline between the two groups. Concerning medication at baseline, there were no differences considering the number of drug-free patients, drug-naïve patients, antipsychotic, antidepressant or anxiolytic treatment between the two groups. The highest use of a mood stabilizer was found in the Toxo+ group (*p* = 0.01).

### 3.2. Comparison of EOS, ELR and ENLR Levels between Cannabis Users and Non-Users in Patients according to the Seropositivy of T. gondii

**Figure 1:**
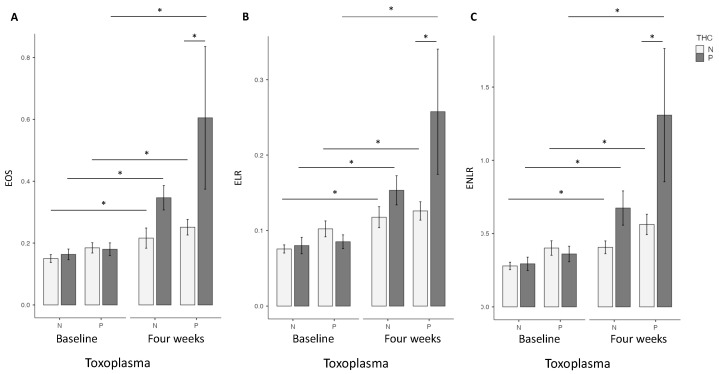
*T. gondii* and cannabis status and Eosinophils (EOS) (**A**), Eosionophils lymphocytes ratio (ELR) (**B**), Eosinophils neutrophils lymphocytes ratio (ENLR) (**C**). No differences were found at baseline concerning these three markers. Higher levels of EOS, ELR and ENLR levels were found in the Toxo+ group at four weeks. Significant increase between baseline and four weeks were found for EOS, ELR and ENLR levels in Toxo+ and Toxo− groups according to cannabis status. THC = tetrahydrocannabinol; N: Negative; P: positive *: *p* < 0.01; test: linear regression; software: Jamovi (Version 2.2).

At baseline, no differences were found for EOS, ELR and ENLR in univariate or multivariate analysis between the two groups (Figure 1).

**Figure 2:**
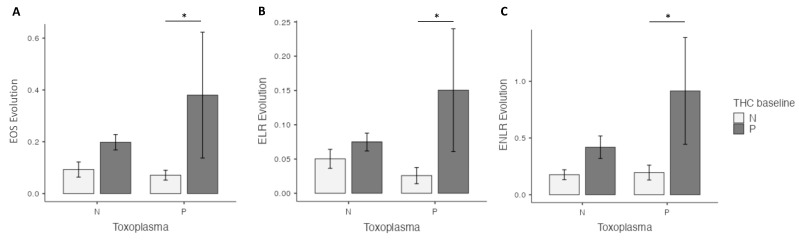
Prospective evaluation of eosinophils (EOS) (**A**), the eosionophils–lymphocytes ratio (ELR) (**B**) and the eosinophils–neutrophils–lymphocytes ratio (ENLR) (**C**) evolution, according to cannabis and *T. gondii* status at four weeks. A higher increase in EOS, ELR and ENLR levels after cannabis cessation versus non-users was found in the Toxo+ group. THC = tetrahydrocannabinol; N: Negative; P: positive. *: *p* < 0.05.

At four weeks, in the univariate analysis, the highest EOS level was found in the cannabis cessation group (*p* = 0.035), but no differences were found for ELR and ENLR levels. However, in the multivariate analysis, highest EOS (*p* = 0.02), ELR (*p* = 0.03) and ENLR levels (*p* = 0.031) were found in the cannabis cessation group (Figure 1). Between baseline and the 4 week-follow up, the THC+ group had a significant increase in EOS (*p* < 0.01), ELR (*p* < 0.01) and ENLR (*p* < 0.01) levels (Figure 1). We found similar results in the group THC−, with a significant increase in EOS (*p* < 0.01), ELR (*p* < 0.01) and ENLR (*p* < 0.01) levels (Figure 1). However, we found that the increase in levels in EOS (*p* = 0.044), and ENLR (*p* = 0.036) were greater in the THC+ group, compared to the THC− group. We also found a trending significant increase in ELR level in the THC+ group (*p* = 0.071) (Figure 2).

### 3.3. Comparison of EOS, ELR and ENLR Levels between Cannabis Users and Non-Users in Patients Seronegative for T. gondii

At baseline, no difference was found between the two groups for EOS, ELR and ENLR, considering univariate or multivariate analysis (Figure 1).

At four weeks, in the univariate analysis, highest EOS (*p* < 0.01), ELR (*p* = 0.036), and ENLR (*p* = 0.043) levels were found in the cannabis cessation group, but these differences did not remain significant in the multivariate analysis (Figure 1). Between baseline and 4 weeks, we found a significant increase in EOS (*p* < 0.01), ELR (*p* < 0.01) and ENLR (*p* < 0.01) levels (Figure 1) in the THC+ group. We found similar results in the THC− group, with a significant increase in EOS (*p* < 0.01), ELR (*p* < 0.01) and ENLR (*p* < 0.01) levels (Figure 1). EOS (*p* = 0.33), ELR (*p* = 0.47) and ENLR (*p* = 0.11) were similar between the two groups (Figure 2).

### 3.4. Interaction between Cannabis and T. gondii Status and EOS, ELR and ENLR

At baseline, we did not find an interaction between cannabis and *T. gondii* status regarding EOS, ELR and ENLR levels.

**Figure 3:**
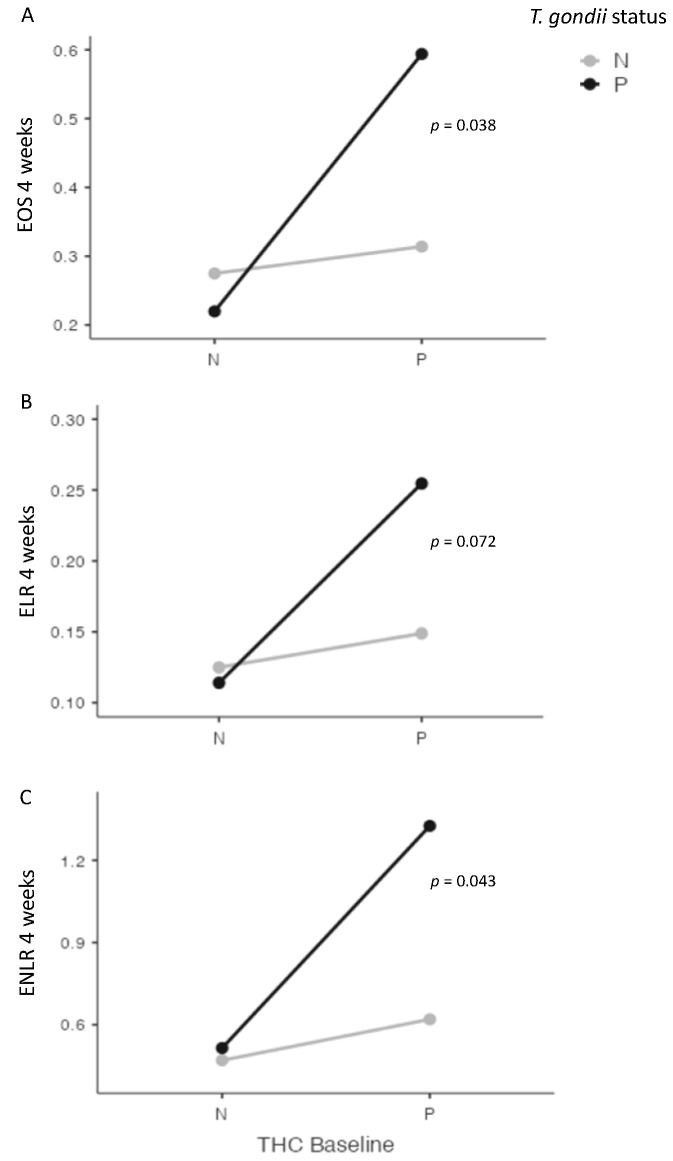
Interactions between cannabis and *T. gondii* status for eosinophils (EOS) (**A**), eosionophils–lymphocytes ratio (ELR) (**B**), and eosinophils–neutrophils–lymphocytes ratio (ENLR) (**C**) at four weeks after cannabis cessation. Significant interactions were found between cannabis and *T. gondii* status for EOS and ENLR levels four weeks after cannabis cessation, with a statistical trend for ELR level. THC = tetrahydrocannabinol; N: Negative; P: positive.

**Figure 4:**
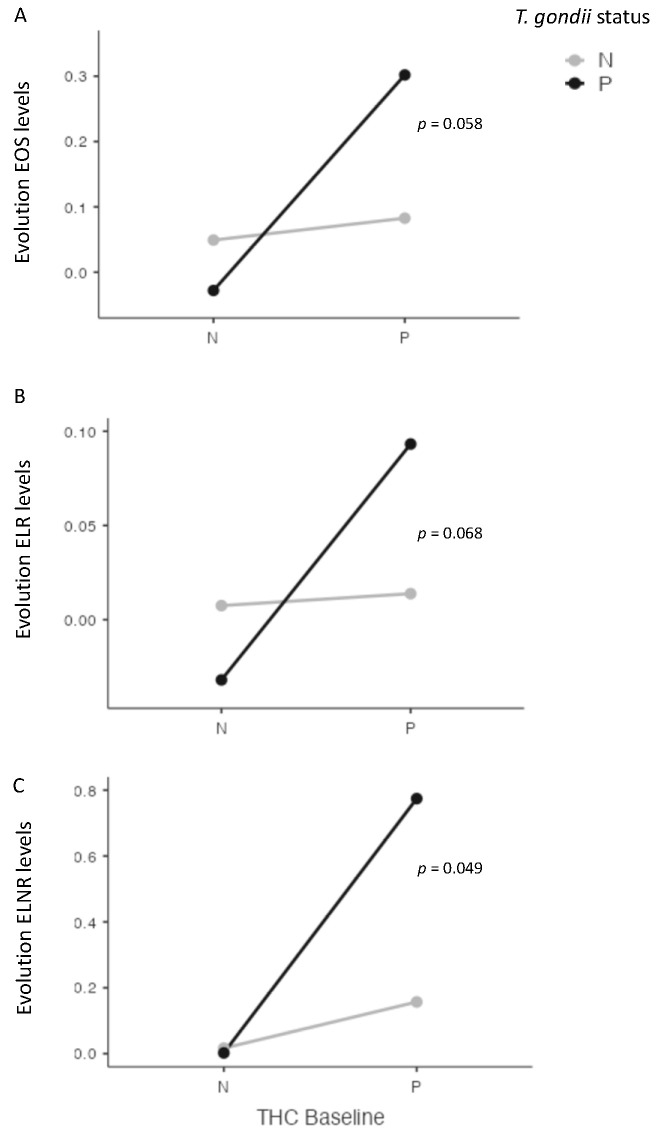
Interactions between cannabis and *T. gondii* status for eosinophils (EOS) (**A**), eosionophils–lymphocytes ratio (ELR) (**B**), and eosinophils–neutrophils–lymphocytes ratio (ENLR) (**C**) between baseline and four weeks after cannabis cessation. A significant interaction was found between cannabis and *T. gondii* status and ENLR level evolution, with a statistical trend for EOS and ELR levels. THC = tetrahydrocannabinol; N: Negative; P: positive.

At four weeks, we found significant interactions between cannabis and *T. gondii* status for EOS (*p* = 0.038), and for ENLR (*p* = 0.043) levels, and a statistical trend for ELR level (*p* = 0.072) (Figure 3). Further, we found a significant interaction between cannabis and *T. gondii* status regarding the evolution between baseline and four weeks of ENLR level (*p* = 0.049), and a statistical trend for the evolution of EOS (*p* = 0.058) and ELR (*p* = 0.068) levels (Figure 4).

### 3.5. Correlation between IgG Antibodies against T. gondii, EOS, ELR, ENLR and Clinical Variables

At baseline, in the THC+ Toxo+ group, a positive correlation was found between IgG antibodies against *T. gondii* and ENLR level (r = 0.492; *p* = 0.024), whereas no correlation was found in the THC- group. In the psychosis subgroup, no correlation was found between IgG antibodies against *T. gondii*, EOS, ELR, ENLR and PANSS score, considering the cannabis status.

At four weeks, no correlation was found between IgG antibodies against *T. gondii* and EOS, ELR, and ENLR, nor with EOS, ELR, and ENLR between baseline and four weeks, regardless of the cannabis status. Patients with psychosis who stopped using cannabis showed a positive correlation between PANSS negative subscale and EOS (r = 0.87; *p* < 0.01) or ELR (r = 0.74; *p* = 0.024) at 4 weeks. Moreover, in the same group, we found a positive correlation between the evolution (between baseline and 4 weeks) of PANSS negative subscale and the evolution of EOS (r = 0.08; *p* = 0.018) or ELR (r = 0.75; *p* = 0.032). In the Toxo+ THC− group, negative correlations were found between EOS level at 4 weeks and PANSS general subscale at 4 weeks (r = −0.44; *p* = 0.018), but also between the evolution of EOS levels and PANSS total score (r = −0.61; *p* < 0.01), PANSS positive subscale score (r = −0.4; *p* = 0.035), PANSS negative subscale score (r = −0.46; *p* = 0.015), and PANSS general subscale score (r = −0.66; *p* < 0.01) at four weeks. A negative correlation was also found between EOS level at 4 weeks and the evolution of PANSS total score (r = −0.66; *p* < 0.01), PANSS positive subscale score (r = −0.56; *p* < 0.01), and PANSS general subscale score (r = −0.65; *p* < 0.01) between baseline and four weeks.

## 4. Discussion

In this study, we found that cannabis cessation in patients with psychiatric disorders was associated with an increase of indirect inflammatory markers such as EOS, ELR and ENLR. Moreover, we found that these disorders seem to be modulated by *T. gondii* seropositivity. Indeed, we found increased levels in EOS, ELR and ENLR at 4 weeks among *T. gondii* positive patients, compared to *T. gondii* negative patients. Furthermore, *T. gondii* status and cannabis cessation were associated with the highest EOS, ELR and ENLR. Finally, we found a positive correlation between negative symptoms and EOS levels at 4 weeks in the group of Toxo+ and THC+ patients.

The role of eosinophils in innate immunity may explain our results. Indeed, eosinophils have traditionally been involved in innate immunity, which contribute to antiparasitic defense or allergy leading to a pro-inflammatory answer [29,36]. In addition, eosinophils participate to the secretion of eosinophil-derived cationic granules, including eosinophil-derived neurotoxin, eosinophil–cationic protein and eosinophil peroxidase, which damage cells when binding charged cell membranes, and thus messing the lipid bilayer and also modifying the activity of enzymes within tissues [37].

Previous studies have already evaluated the impact of cannabis use on inflammatory markers in patients with psychiatric disorders [24,25,26,27,28,29]. Few studies have investigated the specific impact of cannabis cessation on CRP levels and white blood cell counts [26,28]. The lowest levels of CRP were found at baseline in schizophrenic patients, with increased levels of CRP after cannabis cessation, suggesting a restoration of low-grade inflammation after cannabis cessation [26]. This result was confirmed in a larger second study [28]. Despite the fact that CRP levels were not different in our study, we did not find a difference between cannabis users and non users considering EOS ELR and ENLR ratios that had not been tested in previous studies to our knowledge. These results are in line with other studies, which also found no differences between these two populations [24,27]. However, we found again that cannabis cessation was associated with an increase in EOS, ELR and ENLR levels, regarding the *T. gondii* status. This result is in accordance with our previous results, which found that cannabis cessation was associated with increased levels in leucocyte and monocyte [28].

Moreover, we found that inflammatory enhancement after cannabis cessation was modulated by the *T. gondii* infection. Indeed, we found an interaction between *T. gondii* and cannabis cessation for EOS and ENLR levels, and a positive correlation between IgG antibody levels against *T. gondii* and ENLR levels. The link found between *T. gondii* and eosinophils levels is not surprising because eosinophils are thought to be majorly effective to cells in the body’s defense against parasitic infections [34]. Moreover, seropositivity to *T. gondii* has been known to interact with inflammation markers in patients with psychiatric disorders [5,11]. During *T. gondii* infection, cytokines such as IL-6 and IL-27 play a critical role [5]. The first is required for the development of protective immunity against *T. gondii* infection, and the second is crucial for limiting infection-induced inflammatory damage [37]. Eosinophils play a key role in the innate anti-parasite immune response, notably through the secretion of pro-inflammatory cytokines such as IL-6 and Tumor Necrosis Factor α [30,38]. The increase in inflammation found in patients seropositive for *T. gondii* [5,39] could explain the different kinetic of inflammation markers after cannabis cessation between positive or negative patients.

To our knowledge, our study is the first to investigate the link between cannabis consumption and *T. gondii* infection. Endocannabinoid anandamide levels and mRNA transcripts for CB2 receptors are higher during acute episodes of schizophrenia [40,41], during which inflammation is known to be greater [42]. It is also known that chronic *T. gondii* infection, or other brain infections, may increase endocannabinoid levels [43] with neuroprotective effects. We can therefore hypothesize that in patients with psychiatric disorders with latent *T. gondii* infection, cannabis consumption may enhance the anti-inflammatory effect of endocannabinoids.

We found a positive correlation between EOS and ELR levels and PANSS negative subscale only in patients with psychosis who stopped using cannabis and who were seropositive for *T. gondii*. A link between *T. gondii* infection and negative symptoms has been found in patients with schizophrenia [11,44] and, more precisely, when considering alogia (defined by a poverty of speech) [11]. This association can be explained by the decreased neural activity in the ventral striatum and the reduced connectivity in reward-relevant neural circuitry [45] observed in negative symptoms. Our hypothesis is that the negative symptoms are induced by increased inflammation after cannabis cessation, which is modulated by *T. gondii* infection.

Given the high frequency of dual disorders, and, in particular, the comorbidity of addiction with psychiatric disorders [46,47,48], our findings should bring clinical relevance. Indeed, they could partly explain the link between cannabis use and psychiatric disorders, and thus define and target specific, high-risk populations to ensure personalized care. In addition, these results could provide clues for the development of treatments targeting other receptors such as the endocanabinoid system. However, further studies are necessary, notably using specific markers of inflammation (such as cytokines).

Our study has several limitations. Firstly, because of the retrospective design of our study, we are not able to assert our conclusions. In addition, we were unable to obtain precise information on the characteristics of the addictions presented by the patients, as these were not always reported in the clinical records. Indeed, information such as frequency of use, potency of cannabis or exact THC/cannabidiol ratio could be useful to precisely analyse our results. Secondly, the collection of data from medical records may be a source of significant bias. Finally, the absence of evaluation of other markers of inflammation limits the scope of our results. The naturalistic design of this study and its non-interventional approach (notably the use of data only used in routine) partly explain these limitations, and may be confirmed by fully designed studies.

## 5. Conclusions

In conclusion, this study shows that the increase of inflammation after cannabis cessation could be modulated by seropositivity for *T. gondii*, leading to an activation of the innate system highlighted by an over secretion of eosinophils (EOS, ELR and ENLR). Studying the different factors before and after cannabis cessation could be useful to better understand the dual diagnosis between cannabis use disorder and psychiatric disorder and enable future specific treatment or intervention.

## Figures and Tables

**Table 1:** Baseline population characteristics.

		Toxo− (n = 106)	Toxo+ (n = 82)	*p*
Age (years)		31.3 (11.2)	45 (14.7)	**<0.01**
Gender F (%)		55 (51.9)	34 (41.5)	0.16
BMI (kg/m^2^)		23.7 (5.2)	24.1 (4.8)	0.46
Diagnosis (%)	Schizophrenia	30 (28.3)	32 (39)	0.18
	Schizo-affective disorder	10 (9.4)	13 (15.9)	
	FEP	13 (12.3)	6 (7.3)	
	Bipolar Disorder	19 (17.9)	13 (15.9)	
	MDD with psychotic symptoms	5 (4.7)	4 (4.9)	
	MDD without psychotic symptoms	20 (18.9)	14 (17.1)	
	Other Psychotic disorder	2 (1.9)	0 (0)	
	Anxiety disorder	1 (0.9)	0 (0)	
	Personality disorder	6 (5.7)	0 (0)	
Medication at Baseline (%)	Drug-free	63 (59.4)	44 (53.7)	0.43
	Drug-Naïve	36 (34)	17 (20.7)	0.05
	AP	27 (25.5)	26 (31.7)	0.35
	AD	24 (22.6)	14 (17.1)	0.35
	MS	7 (6.6)	15 (18.3)	**0.01**
	Anxiolytic	14 (13.2)	16 (19.5)	0.24
PANSS score *	Total	84.8 (21.2)	87.2 (21.3)	0.52
	Positive	24 (8.7)	24.9 (10.3)	0.78
	Negative	18.5 (8.7)	19.3 (8.8)	0.60
	General	42.3 (9.4)	43 (9.5)	0.69
Smoking status (%)		45 (43.7)	51 (63.7)	**<0.01**
Cannabis status (%)		26 (24.5)	22 (26.8)	0.72

All continuous data were shown as Mean (SD); BMI: Body Mass Index; FEP: First episode psychosis; MDD: Major depressive disorder; AP: Antipsychotic; AD: Antidepressant; MS; Mood Stabilizer; * Only for patients with psychosis.

## Data Availability

The data presented in this study are available on request from the corresponding author.
